# Ecological Validity of Expressed Emotion in Early Psychosis

**DOI:** 10.3389/fpsyt.2019.00854

**Published:** 2019-11-21

**Authors:** Lídia Hinojosa-Marqués, Tecelli Domínguez-Martínez, Thomas R. Kwapil, Neus Barrantes-Vidal

**Affiliations:** ^1^Departament de Psicologia Clínica i de la Salut, Universitat Autònoma de Barcelona, Barcelona, Spain; ^2^Centro de Investigación en Salud Mental Global, Dirección de Investigaciones Epidemiológicas y Psicosociales, Instituto Nacional de Psiquiatría “Ramón de la Fuente Muñiz”, Mexico City, Mexico; ^3^Department of Psychology, University of Illinois at Urbana-Champaign, Champaign, IL, United States; ^4^Departament de Salut Mental, Sant Pere Claver-Fundació Sanitària, Barcelona, Spain; ^5^Centre for Biomedical Research Network on Mental Health (CIBERSAM), Instituto de Salud Carlos III, Barcelona, Spain

**Keywords:** experience sampling, criticism, emotional over-involvement, early psychosis, family environment

## Abstract

Expressed emotion (EE) is an aspect of the family environment that influences the course of multiple forms of psychopathology. However, there is limited research about how EE dimensions [i.e., criticism and emotional over-involvement (EOI)] are expressed in real-world settings. The present study used experience sampling methodology to investigate: 1) the criterion and construct validity of daily-life, momentary measures of criticism and EOI, and 2) the construct and ecological validity of psychometric EE-dimensions as assessed with the self-report Family Questionnaire (FQ). A total sample of 55 relatives (34 relatives of at-risk mental state patients and 21 of first-episode psychosis patients) were prompted randomly six times daily for 1-week to assess their current emotional experiences and cognitive appraisals. Relatives also completed the FQ. Momentary criticism and EOI were significantly associated with the two FQ-EE dimensions respectively, supporting the criterion validity of real-world assessed EE dimensions. As hypothesized, momentary and FQ-EE dimensions were associated with decreased positive affect, as well as with appraisals of less effective coping in daily life. Only momentary EE dimensions were associated with increased momentary negative affect. Partly in contrast with our hypotheses, momentary criticism and FQ-criticism were more consistently related to situational stress and burden than momentary EOI and FQ-EOI. Finally, neither momentary nor FQ-EE dimensions showed distinct patterns of associations with illness attributions. Findings partly support the construct validity of momentary criticism and EOI as well as the construct and ecological validity of the FQ as a sensitive measure of EE dimensions.

## Introduction

The prognostic value of factors identified in the early stages of psychosis (1–3) has renewed the interest in environmental stressors as co-participating factors in the risk, onset, expression, and progression of psychosis (4–7). One of the most significant factors in psychosocial research in psychosis has been expressed emotion (EE) (8), a measure of the family environment used to describe relatives’ attitudes toward an ill family member. High-EE attitudes, particularly criticism and emotional over-involvement (EOI), have been considered the strongest psychosocial predictor of relapse in schizophrenia (9–11).

Although most studies on EE have been carried out in patients with chronic psychotic disorders (12, 13), recent research has focused on the study of EE in the early stages of the disorder (14, 15), in order to improve the understanding of the early development of high-EE and the problematic associated outcomes. Increasing evidence suggests that relatives of patients with first-episode of psychosis (FEP) and at-risk mental state (ARMS) often report high-EE levels (16–19). Moreover, relatives of patients with early psychosis often report high levels of burden, distress, depression, and anxiety related to high levels of EE (20–24). Specifically, there is converging evidence suggesting that EOI is more related to distress and burden than criticism (17, 25–28). Given the large variety of negative outcomes associated with high-EE, both for relatives and patients, it is crucial to examine the psychological underpinnings of EE in early psychosis without the bias created by the chronic course of the illness and relatives’ long-term burden.

The attributional model of EE posits that relatives’ beliefs about the causes of the patients’ illness are linked to relatives’ emotional attitudes toward patients (29). It seems that critical relatives are more likely to perceive symptoms as controllable by patients, even at recent-onset psychosis stages (30, 31). Consequently, they attempt to reduce the undesired behaviors through critically persuading or coercing the patient. In contrast, overinvolved relatives tend to attribute symptoms to external factors that are beyond the patients’ control (32, 33). However, they may also perceive patients’ symptoms under their own control (34) and even can report high levels of guilt and/or self-blame attributions (35, 36). This in turn makes them more likely to exhibit intrusive and/or self-sacrificing attitudes. An alternative explanatory model of EE proposes that high-EE attitudes may represent a maladaptive attempt to cope with the stress of caring for an impaired ill relative; thus, EE behaviors could be deemed as maladaptive coping strategies used to reduce the perceived stress related to the caregiving role (25, 37). In fact, relatives of patients with schizophrenia with high EE perceive their ability to cope as less effective and more impaired than those with low-EE (38). Also, emotion-focused strategies, such as avoidant coping, have been related to high-EE attitudes in relatives of FEP patients (22, 37).

Few studies have examined EE in early psychosis patients, and these have typically relied on retrospective reports. Furthermore, the vast majority of studies have not considered how EE is displayed within the natural family environment. This is a limitation given that EE is conceptualized within an interactional framework (12, 39, 40). Therefore, it is crucial to examine how EE is expressed within the contexts of relatives’ interactions with patients and in relation to daily appraisals and caregivers’ subjective states in real-world settings.

The construct and ecological validity of EE has been supported by research showing that high-EE relatives tend to be more critical and intrusive in their direct transactions with patients than low-EE relatives (41–43). However, most studies have assessed family interactions as a function of global EE, without considering that criticism and EOI involve different underlying appraisals and may be associated with differential patterns of behaviors (44–46). Specifically, relatives high on the criticism dimension manifest critical attitudes at a behavioral level (47–49) and tend to offer demeaning statements in parent-patient interactions (50). However, findings supporting the construct validity of EOI have been less consistent (50, 51). Some studies indicate that high-EOI relatives make intrusive (52) or ambiguous statements (53), whereas others report that they exhibit high rates of positive and supportive statements in interaction studies (54). These studies offer valuable information of the behavioral correlates of EE in laboratory settings. However, as these paradigms focus on objectively defined family interactional tasks (e.g., interactions lasting 10 min or longer), they are unable to capture how EE components are expressed in daily life and relate to the wide variety of caregiver’s subjective states within the natural family environment.

Unlike previous research, the current study employed experience sampling methodology (ESM) to examine the daily life expression of EE. ESM provides several benefits compared to conventional laboratory or clinic-based assessment paradigms (e.g., 55): 1) it enhances ecological validity because it allows to evaluate participants in their daily environment, 2) it captures participant’s experience in the moment, thus minimizing retrospective bias, and 3) allows for an examination of the context in which the experience is occurring. To the best of our knowledge, the only study that has used an ecologically valid methodology to examine family interactions reported that relatives’ high-EE status did not influence relatives’ affect when relatives were in contact with patients (56). Although this work provided an ecologically valid insight on EE dynamics, it did not specifically measure the expression of EE dimensions in daily life.

Therefore, the general purpose of this study was to test the ecological validity of the criticism and EOI dimensions in a sample of relatives of early psychosis patients. To this end, the first aim of the current study was to analyze the criterion validity of momentary criticism and EOI (i.e., as measured with ESM) by examining their association with the EE-dimensions of the Family Questionnaire (FQ) (57), a widely used psychometric measure of EE. Regarding this first aim, it was predicted that the analogous momentary and psychometric indicators of criticism and EOI would be significantly and more strongly related to each other than to the other dimension.

The second aim was to examine the construct validity of momentary criticism and EOI, as well as the construct and ecological validity of psychometric EE dimensions by examining their associations with emotional, cognitive, and interpersonal behaviors occurring in the flow of daily life. We expected that momentary and psychometric criticism and EOI would show both common and distinctive correlates:

- Both EE dimensions (momentary and psychometric) were expected to be related to increased negative affect and decreased positive affect, appraisals of less effective coping, and momentary reports of increased situational stress and burden.
- Based on the attributional model of EE, different correlates were expected for criticism and EOI in relation to illness attributions. Momentary and FQ-criticism were predicted to be associated with attributions of control toward the patient, whereas momentary EOI and FQ-EOI were expected to be related to attributions of personal control over the disorder in daily life.

Additionally, we explored whether criticism and EOI, as measured by momentary and psychometric self-reports, showed differential associations with negative and positive appraisals about the self, positive appraisals about the patient, as well as with negative and positive appraisals about patients’ behaviors in situations of direct and/or recent contact with the patient. Given the paucity of previous research the following hypotheses were exploratory. It was expected that high criticism would be related to negative appraisals about the patient, whereas EOI would show some associations with positive appraisals of the patient.

## Materials and Methods

### Participants and Procedure

The present study is embedded in a larger longitudinal study carried out in three Mental Health Centres in Barcelona (Spain) within the Sant Pere Claver-Early Psychosis Program (SPC-EPP) (58). A total of 55 relatives of early psychosis patients (34 from ARMS and 21 from FEP patients), recruited in the SPC-EPP, were included in this study. An additional six participants were enrolled in the study and completed the questionnaires but were omitted from the analyses due to failing to complete the ESM protocols.

Relatives were referred to the study by their respective affected family members (i.e., early psychosis patients who were already participating in the study). Patients were informed of the relatives’ study and asked to name the person to whom they have a significant/close relationship. After getting the consent of the patient, the relative was contacted and was asked to participate into the study. The recruited relatives were those who had the most regular contact and/or the most significant relationship with the patient. Relatives were predominantly female (67.3%), specifically patients’ mothers (63.6%), with the remaining caregivers being fathers (25.5%), partners, or siblings (11%). Mean age of the relatives was 50.7 years old (*SD* = 8.96). Most relatives lived with the patient (85.5%). Participants completed an average of 28.5 (*SD* = 11.4) usable ESM questionnaires (67.8%).

Patients had to meet ARMS criteria as assessed by the Comprehensive Assessment of At-Risk Mental States (CAARMS) (59) and/or the Schizophrenia Proneness Instrument Adult-Version (SPI-A) (60). FEP patients met DSM-IV-TR criteria (61) for any psychotic disorder or affective disorder with psychotic symptoms as established by the Structured Clinical Interview for DSM-IV (SCID-I) (62). All relatives provided written informed consent to participate. The project was developed in accordance with the Code of Ethics of the World Medical Association (Declaration of Helsinki). Ethical approval for the study was obtained from the local ethics committee.

### Measures

Relatives completed the FQ (57), a well-established instrument to measure EE. The FQ which consists of two 10-item subscales (criticism and EOI), with items answered on a four-point scale ranging from “never/very rarely” to “very often.” The internal consistency (Cronbach’s alpha) of the scores for the subscales in our sample was good, 0.89 for criticism and 0.86 for EOI.

ESM data were collected with personal digital assistants [PDAs; n = 16 (29%)], digital wristwatch and booklet [n = 25 (45%)], and mobile devices [n = 14 (26%)]. The response rates (percentage of completed ESM questionnaires) did not significantly differ among the three methods employed, which were, respectively, 56.4, 75.9, and 66.8% (F = 2.69; *p* = 0.077). Note that numerous studies indicate that these methods produce comparable data in terms of quantity and quality (63, 64). Participants were signaled randomly six times daily (between 11 a.m. and 22 p.m.) for 1 week to complete brief questionnaires. When prompted by the signal, the participants had 5 min to initiate responding. After this time interval or the completion of the questionnaire, the PDA or mobile device became inactive until the next signal. Each questionnaire required 2 min to complete. All ESM items were answered on seven-point scales ranging from “not at all” to “very much,” except for three yes/no items (“Are you with he/she right now?,” “Since the last beep, did you have contact with he/she,” “Right now, I wish he/she was here”).

The ESM questionnaire included items that inquired about the following domains: 1) momentary criticism; 2) momentary EOI; 3) affect in the moment; 4) appraisals of effective coping; 5) appraisals about the current situation; 6) appraisals of burden; 7) illness attributions; 8) appraisals related to the self; 9) positive appraisals about the patient; and 10) a variety of appraisals that are only prompted if there is direct and/or recent contact with the patient. The ESM items inquiring about “appraisals in situations of direct and/or recent contact with the patient” were only administered when relatives endorsed either of the items: “Are you with he/she right now?” or “Since the last beep, did you have contact with he/she.” The rest of the ESM items were always administered.

The momentary criticism index was created using the following 4 items: “I feel exhausted by (the patient),” “I feel disappointed by (the patient),” “I feel angry with (the patient),” and “It is difficult to deal with (the patient)” (Cronbach’s α = 0.83). Momentary EOI was assessed with the item “I am worried about (the patient).” Momentary EE items were developed on the basis of construct definitions and the items of the FQ (57). Summary indices were also computed for negative affect (NA) (Cronbach’s α = 0.81), positive affect (PA) (Cronbach’s α = 0.87), and relatives’ positive appraisals about patients’ behaviors in situations of direct and/or recent contact with the patient (Cronbach’s α = 0.81). **Table 1** displays the ESM items and indices.

**Table 1:** Relatives’ ESM questionnaire and summary indices.

Questionnaire	Summary indices
1. Right now, I feel happy.	
2. Right now, I feel sad.	Indices are computed as the means of the items indicated.
3. Right now, I feel I can cope with things well.	Momentary criticism: 20, 21, 22, and 24
4. Right now, it is difficult to concentrate or make decisions.	Negative affect: 2, 7, and 8
5. Right now, I feel relaxed.	Positive affect: 1, 5
6. Right now, I feel lonely.	Positive appraisals about patients’ behaviors: 31, 32, and 34
7. Right now, I feel irritable.	
8. Right now, I feel anxious.	
9. Right now, I feel hopeful.	
10. Right now, I feel guilty.	
11. Right now, I have difficulty controlling my thoughts and emotions.	
12. Right now, I like what I am doing.	
13. Right now, I feel tired.	
14. Right now, I don’t feel physically well.	
15. Right now, I feel supported.	
16. My current situation is positive.	
17. My current situation is stressful.	
18. Right now, I feel happy with he/she.	
19. Right now, I am worried about he/she.	
20. Right now, I feel exhausted by he/she.	
21. Right now, I feel disappointed by he/she.	
22. Right now, I am angry with he/she.	
23. Right now, I feel close to he/she.	
24. Right now, it is difficult to deal with he/she.	
25. Right now, I feel that he/she doesn’t make an effort to be well.	
26. Right now, he/she is a burden to me.	
27. Right now, I feel he/she cannot function without me.	
28. ¿Are you with he/she right now? *[If YES selected: Q31–Q36/If NO selected: Q29]*	
29. Since the last beep, did you have contact with he/she? *[If YES selected: Q31–36/If NO selected: Q30 and END of SURVEY]*	
30. Right now, I wish he/she was here.	
31. Right now, he/she is functioning well.	
32. Right now, he/she is in a good mood.	
33. Right now, he/she is being disruptive.	
34. Right now, it is good to have he/she around.	
35. Right now, he/she makes me feel exhausted.	
36. Right now, he/she is a burden to me.	

### Statistical Analysis

Pearson correlations were computed to explore the association of momentary EE constructs (i.e., criticism and EOI) with psychometric EE dimensions (FQ) using the Statistical Package for Social Sciences (SPSS), Version 22.0. The effect size of the correlations was interpreted following Cohen’s (65) guidelines (correlations of 0.10 indicate small effect sizes, 0.30 indicate medium effect sizes, and 0.50 indicate large effect sizes).

The statistical analyses involving the ESM data were conducted with Mplus 6 (66) ESM data have a multilevel structure in which ESM ratings (level 1 data) are nested within participants (level 2 data). Multilevel or hierarchical linear modeling techniques are a standard approach for the analysis of ESM data (67, 68). Level 1 predictors were group-mean centered, level 2 predictors were grand-mean centered, and parameter estimates were calculated using robust standard errors. Two types of multilevel analyses were conducted in the present study. Firstly, a series of multilevel regressions were conducted to test the impact of momentary criticism and EOI (level 1 predictors) on emotional, cognitive, and interpersonal experiences in daily life. Secondly, multilevel regressions were performed to explore the impact of FQ-EE dimensions (level 2 predictors) on ESM domains in daily life (level 1 dependent measures).

## Results

### Associations Between Momentary Expressed Emotion and Family Questionnaire-Expressed Emotion Dimensions

Momentary criticism and momentary EOI were significantly correlated in the present sample (*r* = 0.59, *p* = 0.000, LLCI = 0.425, ULCI = 0.719). Pearson’s correlations revealed strong associations between momentary criticism and FQ-criticism (*r* = 0.66, *p* = 0.000, LLCI = 0.452, ULCI = 0.814), as well as between momentary EOI and FQ-EOI (*r* = 0.51, *p* = 0.000, LLCI = 0.305, ULCI = 0.675). Following Cohen (65), effect sizes were of large magnitude. Significant associations were also found between momentary criticism and FQ-EOI (**r** = 0.42, *p* = 0.002, LLCI = 0.175, ULCI = 0.623) as well as between momentary EOI and FQ-criticism (*r* = 0.45, *p* = 0.001, LLCI = 0.242, ULCI = 0.640), both medium effect sizes.

### Associations Between Momentary Expressed Emotion and Emotional, Cognitive, and Interpersonal Experiences in Daily Life

**Table 2** presents the direct effects of the momentary EE dimensions on relatives’ daily life experiences. As expected, momentary criticism and EOI were associated with increased NA and decreased PA in daily life. Additionally, both momentary EE dimensions were related with decreased appraisals of effective coping in everyday life.

**Table 2:** Direct effects of momentary expressed emotion dimensions on relatives’ daily life experiences.

Level 1 criterion	Level 1 predictors							
	Momentary criticism	*P*	95% CI		Momentary EOI	*P*	95% CI	
			Lower	Upper			Lower	Upper
**Affect in the moment**								
Negative affect index	0.440 (SE = 0.103)	0.000	0.238	0.642	0.092 (SE = 0.028)	0.001	0.037	0.147
Positive affect index	−0.399 (SE = 0.081)	0.000	−0.558	−0.267	−0.150 (SE = 0.030)	0.000	−0.208	−0.091
**Appraisals of effective coping**								
Right now, I feel I can cope with things well.	−0.173 (SE = 0.056)	0.002	−0.284	-0.063	−0.070 (SE = 0.034)	0.037	−0.136	−0.004
**Appraisals about the current situation**								
My current situation is positive.	−0.214 (SE = 0.070)	0.002	−0.351	−0.076	−0.073 (SE = 0.030)	0.013	−0.131	−0.015
Right now, I like what I am doing.	−0.190 (SE = 0.056)	0.001	−0.300	−0.080	−0.057 (SE = 0.028)	0.044	−0.112	−0.001
My current situation is stressful.	0.366 (SE = 0.062)	0.000	0.245	0.468	0.033 (SE = 0.030)	0.271	−0.026	0.093
**Appraisals of burden**								
Right now, he/she is a burden to me.	0.321 (SE = 0.097)	0.001	0.131	0.512	0.112 (SE = 0.039)	0.004	0.036	0.188
**Illness attributions**								
*Attributions of patients’ control over the disorder*								
Right now, I feel that he/she doesn’t make an effort to be well.	0.473 (SE = 0.102)	0.000	0.273	0.673	0.157 (SE = 0.042)	0.000	0.075	0.238
*Attributions of relatives’ control over the disorder*								
Right now, I feel that he/she cannot function without me.	0.167 (SE = 0.070)	0.017	0.030	0.304	0.090 (SE = 0.030)	0.002	0.032	0.149
**Appraisals related to the self**								
*Positive appraisals*								
Right now, I feel hopeful.	−0.077 (SE = 0.050)	0.120	−0.175	0.020	−0.036 (SE = 0.019)	0.062	−0.075	0.002
Right now, I feel supported.	−0.157 (SE = 0.063)	0.013	−0.281	−0.034	−0.055 (SE = 0.035)	0.110	−0.123	0.012
*Negative appraisals*								
Right now, I feel lonely.	0.211 (SE = 0.151)	0.160	−0.084	0.507	0.063 (SE = 0.028)	0.026	0.008	0.118
Right now, I feel guilty.	0.089 (SE = 0.062)	0.148	−0.032	0.210	0.037 (SE = 0.024)	0.125	−0.010	0.084
**Positive appraisals about the patient**								
Right now, I feel happy with he/she.	−0.605 (SE = 0.128)	0.000	−0.856	−0.354	−0.183 (SE = 0.051)	0.000	−0.283	−0.082
Right now, I feel close to he/she.	−0.324 (SE = 0.094)	0.001	−0.509	−0.139	−0.056 (SE = 0.026)	0.031	−0.107	−0.005
**Appraisals in situations of direct and/or recent contact with the patient**								
*Negative appraisals about patients’ behaviors*								
Right now, he/she is being disruptive.	0.498 (SE = 0.072)	0.000	0.357	0.639	0.198 (SE = 0.039)	0.000	0.121	0.274
Right now, he/she makes me feel exhausted.	0.462 (SE = 0.082)	0.000	0.301	0.623	0.116 (SE = 0.048)	0.017	0.021	0.211
Right now, he/she is a burden to me.	0.264 (SE = 0.154)	0.085	−0.037	0.566	0.044 (SE = 0.053)	0.407	−0.060	0.148
*Positive appraisals about patients’ behaviors index*	−0.359 (SE = 0.052)	0.000	−0.461	−0.257	−0.181 (SE = 0.036)	0.000	−0.251	−0.112

EE,expressed emotion; EOI,emotional over-involvement; SE,standard error; 95% CI,95% confidence interval.

In terms of appraisals about the current situation, momentary criticism and EOI were associated with a decreased enjoyment of current activities and perceiving the current situation as less positive. Momentary criticism was associated with reports that the current situation was stressful, whereas contrary to expectations, momentary EOI was not. In relation to burden, both momentary EE domains showed significant associations with increased appraisals of feeling burdened by the patient. Also, contrary to our hypotheses, no differential associations emerged for the momentary EE dimensions in relation to illness attributions. Both momentary EE dimensions were significantly associated with attributions of patients’ control over the disorder as well as with attributions of relatives’ personal control over the disorder in daily life.

Regarding negative and positive appraisals related to the self, momentary criticism was related to appraisals of feeling less supported, whereas momentary EOI was associated with feelings of being lonely. No associations were found between momentary EE and appraisals of hope and guilt. In relation to relatives’ positive appraisals about the patient, momentary criticism, and EOI were associated with expressing decreased feelings of happiness in relation to the patient and less reports of feeling emotionally close to the patient.

As for relatives’ appraisals in situations of direct and/or recent contact with the patient, momentary criticism and EOI were associated with current appraisals of feeling exhausted by the patient as well as with perceiving the current patient’s behavior as disruptive. There were no associations between momentary EE and appraisals of burden. Moreover, both momentary EE dimensions were inversely related to positive appraisals about patients’ behaviors when relatives were interacting and/or had recently interacted with the patient.

### Impact of Psychometric Expressed Emotion Dimensions on Emotional, Cognitive, and Interpersonal Experiences in Daily Life

FQ criticism and EOI scores were significantly correlated in the present sample (*r* = 0.72, *p* = 0.000, LLCI = 0.576, ULCI = 0.823). **Table 3** presents the direct effects of FQ-EE dimensions on relatives’ daily life experiences. FQ-criticism was related with the momentary criticism index and its four individual items. Specifically, FQ-criticism was associated with increased reports of feeling exhausted by the patient, disappointed and angry with the patient, as well as with an increased perception of difficulties for dealing with the patient. FQ-criticism was also related to momentary EOI. On the other hand, FQ-EOI was related to momentary EOI that is, to increased reports of worry about the patient. In addition, FQ-EOI was associated with the momentary criticism index, specifically with feeling exhausted by the patient and perceiving difficulties in dealing with the patient, but not with feeling disappointed or angry with the patient.

**Table 3:** Direct effects of psychometric expressed emotion dimensions on relatives’ daily life experiences.

Level 1 criterion	Level 2 predictors							
	FQ-criticism	*P*	95% CI		FQ-EOI	*P*	95% CI	
			Lower	Upper			Lower	Upper
**Momentary EE**								
***Momentary criticism-index***	0.091 (SE = 0.014)	0.000	0.064	0.118	0.062 (SE = 0.018)	0.001	0.026	0.097
Right now, I feel exhausted by he/she.	0.091 (SE = 0.018)	0.000	0.056	0.126	0.093 (SE = 0.018)	0.000	0.057	0.129
Right now, I feel disappointed by he/she.	0.081 (SE = 0.021)	0.000	0.039	0.122	0.045 (SE = 0.024)	0.065	−0.003	0.093
Right now, I am angry with he/she.	0.048 (SE = 0.014)	0.001	0.020	0.076	0.022 (SE = 0.018)	0.226	−0.013	0.057
Right now, it is difficult to deal with he/she.	0.144 (SE = 0.021)	0.000	0.103	0.186	0.088 (SE = 0.032)	0.006	0.626	1.166
***Momentary EOI***	0.117 (SE = 0.028)	0.000	0.062	0.172	0.141 (SE = 0.028)	0.000	0.086	0.197
**Affect in the moment**								
Negative affect-index	0.043 (SE = 0.025)	0.079	−0.005	0.092	0.045 (SE = 0.025)	0.076	−0.005	0.094
Positive affect-index	−0.065 (SE = 0.024)	0.007	−0.112	−0.018	−0.074 (SE = 0.025)	0.003	−0.114	0.025
**Appraisals of effective coping**								
Right now, I feel I can cope with things well.	−0.075 (SE = 0.017)	0.000	−0.108	−0.043	−0.070 (SE = 0.018)	0.000	−0.105	−0.035
**Appraisals about the current situation**								
My current situation is positive.	−0.071 (SE = 0.019)	0.000	−0.108	−0.034	−0.083 (SE = 0.022)	0.000	−0.125	−0.040
Right now, I like what I am doing.	−0.032 (SE = 0.020)	0.104	−0.070	0.007	−0.038 (SE = 0.020)	0.060	−0.078	0.002
My current situation is stressful.	0.082 (SE = 0.023)	0.000	0.037	0.128	0.065 (SE = 0.030)	0.031	0.006	0.124
**Appraisals of burden**								
Right now, he/she is a burden to me.	0.117 (SE = 0.048)	0.015	0.023	0.211	0.090 (SE = 0.048)	0.058	−0.003	0.012
**Illness attributions**								
***Attributions of patients’ control over the disorder***								
Right now, I feel that he/she doesn’t make an effort to be well.	0.091 (SE = 0.024)	0.000	0.044	0.138	0.054 (SE = 0.028)	0.050	0.000	0.108
***Attributions of relatives’ control over the disorder***								
Right now, I feel that he/she cannot function without me.	0.134 (SE = 0.032)	0.000	0.070	0.198	0.135 (SE = 0.032)	0.000	0.072	0.198
**Appraisals related to the self**								
*Positive appraisals*								
Right now, I feel hopeful.	−0.041 (SE = 0.017)	0.017	−0.074	−0.007	−0.046 (SE = 0.019)	0.016	−0.083	−0.008
Right now, I feel supported.	−0.049 (SE = 0.030)	0.099	−0.106	0.009	−0.048 (SE = 0.027)	0.074	−0.101	0.005
*Negative appraisals*								
Right now, I feel lonely.	0.053 (SE = 0.032)	0.093	−0.009	0.115	0.047 (SE = 0.030)	0.115	−0.011	0.104
Right now, I feel guilty.	0.089 (SE = 0.042)	0.034	0.007	0.171	0.106 (SE = 0.040)	0.008	0.027	0.184
**Positive appraisals about the patient**								
Right now, I feel happy with he/she.	−0.083 (SE = 0.026)	0.001	−0.134	−0.033	−0.099 (SE = 0.028)	0.000	−0.154	−0.044
Right now, I feel close to he/she.	−0.065 (SE = 0.027)	0.016	−0.117	−0.012	−0.044 (SE = 0.027)	0.100	−0.097	0.009
**Appraisals in situations of direct and/or recent contact with the patient**								
***Negative appraisals about patients’ behaviors***								
Right now, he/she is being disruptive.	0.117 (SE = 0.022)	0.000	0.074	0.160	0.072 (SE = 0.028)	0.010	0.017	0.128
Right now, he/she makes me feel exhausted.	0.088 (SE = 0.025)	0.000	0.039	0.136	0.083 (SE = 0.025)	0.001	0.035	0.132
Right now, he/she is a burden to me.	0.012 (SE = 0.041)	0.764	−0.068	0.093	−0.032 (SE = 0.048)	0.513	−0.126	0.063
***Positive appraisals about patients’ behaviors index***	−0.066 (SE = 0.017)	0.000	−0.099	−0.033	−0.054 (SE = 0.018)	0.002	−0.090	−0.019

FQ, Family Questionnaire; EE, expressed emotion; EOI, emotional over-involvement; SE, standard error; 95% CI,95% confidence interval.

Although none of two FQ-EE dimensions showed significant relationships with momentary NA, both were inversely related to momentary PA. Furthermore, both dimensions were related to decreased appraisals of effective coping in daily life.

In terms of situational appraisals, both dimensions were associated with perceiving situations as less positive and more stressful. No associations were found with appraisals of enjoyment of current activities. Unlike FQ-EOI, FQ-criticism was associated with increased momentary reports of feeling burdened by the patient. Regarding illness attributions, both dimensions were associated with attributions of relatives’ personal control over the disorder. However, only FQ-criticism was associated with increased attributions of patients’ control over the disorder as expected.

As for negative and positive appraisals related to the self, both dimensions were associated with guilt and diminished hopefulness, but were unassociated with feeling supported or lonely. Both dimensions were associated with decreased feelings of happiness with the patient. However, only FQ-criticism was associated with feeling less close to the patient.

No differential associations were found in relation to the appraisals in situations of direct and/or recent contact with the patient. Both FQ-criticism and FQ-EOI displayed significant relationships with increased reports of feeling exhausted by the patient as well as with perceiving the patient’s behavior as disruptive. No associations were found between EE and burden. Moreover, both FQ dimensions were inversely related to positive appraisals about patients’ behaviors.

## Discussion

To the best of our knowledge, this is the first study to explore how EE dimensions, as measured by momentary and psychometric self-reports, are expressed in daily life by using ESM in a sample of caregivers of patients with early psychosis. Consistent with our hypotheses, momentary criticism and FQ-criticism, as well as momentary EOI and FQ-EOI, displayed significant associations of a large magnitude, thus providing support for the criterion validity of momentary EE dimensions. Furthermore, findings showed that both momentary EE and FQ-EE dimensions were significantly and meaningfully associated with real-world experiences pertaining to psychological domains that have been previously related to EE in retrospective and psychometric studies that are critical to the definition of EE from a theoretical standpoint. Overall, the findings partly support the construct validity of momentary criticism and EOI as well as the construct and ecological validity of the FQ.

The strong association between analogous EE momentary and psychometric dimensions supported the criterion validity of momentary criticism and EOI assessments. However, there were also significant associations between momentary criticism and FQ-EOI, as well as between momentary EOI and FQ-criticism—although of a medium, not large, magnitude. Hence, momentary criticism and momentary EOI appear to be relatively non-specific indicators of each respective EE dimension (i.e., criticism and EOI). A detailed examination of the relationship between FQ-EOI and the four individual items comprised in the momentary criticism index partially supported the discriminant validity of the FQ-EOI dimension, as FQ-EOI was associated to feeling exhausted by the patient and perceiving difficulties for dealing with the patient, but not with the most representative appraisals of the momentary criticism index (i.e., feeling disappointed and angry with the patient). Overall, our findings concur with previous research indicating significant associations between the EE dimensions (69, 70) which contrasts with previous suggestions posing that they are uncorrelated and represent independent constructs (44–46).

Overall, the results regarding the daily life expression of criticism and EOI dimensions partly confirmed our hypotheses. Momentary criticism and EOI were related to increased NA and decreased PA, consistent with previous work indicating that early psychosis relatives often report high levels anxiety and depression associated to high levels of EE (20, 24, 26, 27). However, FQ-EE dimensions were only associated to decreased PA in daily life, suggesting that momentary measure of EE is more sensitive for capturing NA experiences. Furthermore, both momentary and psychometric EE indicators were related to appraisals of less effective coping in daily life. This finding is in accordance with previous results from the schizophrenia literature showing that relatives with high-EE perceive their coping ability as poorer than those with low-EE (38), and further supports the assumption that EE could be deemed as a maladaptive coping strategy used in an attempt to reduce the perceived stress related to the caregiving role (25, 37).

Regarding relatives’ appraisals about the situation, results showed that FQ and momentary criticism and EOI were associated with reports that the current situation was less positive. However, unlike FQ-EE dimensions, momentary EE dimensions also displayed associations with expressing decreased enjoyment regarding current activities. Consistent with our results, Hooley and Hiller (71) found that relatives of schizophrenia patients with high-EE reported reduced satisfaction about their individual activities compared to low-EE relatives. It is likely that the momentary nature of ESM allows to capture with higher sensitivity the rewarding capacity of situations encountered in the flow of daily life compared to retrospective inventories.

In line with our hypotheses, momentary criticism and FQ-criticism were related to daily appraisals of situational stress and burden. However, momentary EOI and FQ-EOI showed discrepancies in their association to burden and stress. FQ-EOI, but not momentary EOI, was associated with increased appraisals of situational stress, whereas only momentary EOI was related with increased appraisals of feeling burdened by the patient. Thus, criticism had a clearer association with situational stress and burden than EOI. Overall, these results seem to be partially consistent with previous early psychosis findings indicating an association of EE with relatives’ distress and/or burden (20, 22, 24) but do not replicate previous early psychosis research suggesting that EOI is more strongly related to distress and burden than criticism (17, 25–27).

Partly in contrast with our hypotheses, a distinct pattern of associations was not observed for momentary and FQ-EE dimensions in relation to illness attributions. As expected, momentary criticism and FQ-criticism were associated with increased attributions of patients’ control over the disorder. This result agrees with previous findings in early psychosis (30, 31) and is consistent with the attributional model of EE (29). However, momentary EOI was also associated with attributions of control over the disorder by the patient. This seems to be partly incongruent with the attributional model of EE referred above, which posits a specific relationship between criticism and attributions regarding patients’ ability to control their behaviors. Notwithstanding, EOI has also been related with attributions of control by the patient in the early stages of psychosis (20). On the other hand, and in line with previous studies relating EOI with relatives’ self-control attributions (34), both momentary EOI and FQ-EOI were related to attributions of relatives’ control over the disorder. However, in contrast with our hypotheses, both momentary criticism and FQ-criticism were also related to self-control attributions. It is attractive to speculate that at the early stages of the disorder, when there is great confusion and still a low level of knowledge about the disorder, a great majority of relatives still exhibit low-defined illness attributions. Thus, relatives may believe that they can control the disorder by themselves and, at the same time, that patients can have a significant control over their behavior, which may explain the finding of a lack of differential patterns between illness attributions and EE attitudes. As the disorder progresses, relatives may entrench more defined illness attributions which, in turn, delineate more specific behavioral reactions. Moreover, it is likely that the high-emotional impact of early psychosis on caregivers leads them to a low understanding of their own feelings (i.e., diminished emotional clarity). Presumably, low levels of emotional clarity may affect the way they appraise patients’ behaviors thus provoking relatives’ cognitive ambivalence regarding the control of the disorder. Thus, the attributional model of EE based on schizophrenia samples should be tailored to the developmental specificities of early psychosis (20). This would require integrating the pivotal role of emotional factors influencing the psychological experience of relatives in at-risk and onset stages of the disorder.

Regarding positive and negative appraisals related to the self, our results showed that, unlike momentary EE, both FQ-EE dimensions were associated with increased reports of feeling guilt in daily life. Previous studies have reported associations between relatives’ guilt related to the patient’s illness and EOI behaviors (35, 36). However, Wasserman et al. (72) also found relatives’ guilt/self-blame to be associated with high-EE overall status and posited that relatives may defend against the experience of blaming themselves by putting the blame onto the patient in a critical manner or by behaving in an emotionally over-involved way to repair their wrongdoing. Furthermore, unlike momentary EE, both FQ-EE dimensions were associated to decreased reports of hopefulness. Hopefulness has not been studied in relation to EE, although it has been conceived as crucial in the process of coping with a psychiatric disorder in a close family member (73, 74). Finally, only momentary criticism was related to appraisals of feeling less supported, whereas momentary EOI was associated with feelings of being lonely. In essence, these appraisals could be conceived as indicators of perceived social support. Of note, recent research has found EE to be related with a decreased perception of social support in schizophrenia relatives (75).

In regard to the association of criticism and EOI with daily positive appraisals about the ill relative, both momentary and FQ-EE dimensions were related to decreased happiness regarding their relative. Also, both momentary EE domains and FQ-criticism (but not FQ-EOI) were associated with reports of feeling less close to the patient. Given that EOI is characterized by the expression of extreme emotional closeness with the patient (i.e., overidentification) (76), it is not surprising that FQ-EOI was not related to decreased feelings of closeness. The relationship between momentary EOI and appraisals of decreased closeness might indicate that our measure of EOI, based on the core element of “worrying” but restricted to it, has not been sufficient to capture all the nuances of this construct and/or that the number of daily-life assessments was not extensive enough to capture sufficient variance in such sensitive assessment regarding the parental bond.

As for relatives’ appraisals about the ill relative when relatives were in direct contact and/or had had recent contact with the patient, both momentary and FQ-EE dimensions were related with decreased positive appraisals about patients’ behaviors as well as with increased reports of feeling exhausted by the patient and/or with perceiving the patient’s behavior as disruptive in the current situation. This is consistent with findings that relatives of schizophrenia patients holding highly critical or emotionally overinvolved attitudes tend to show lower levels of accepting behavior (77) and make more negative statements (e.g., criticism, statements of disagreement) during face-to-face interactions with patients than do low-EE relatives (42, 43, 78). Also, our results concur with previous studies indicating that criticism is related to “belittling and blaming” statements (53) and/or with statements of disgust and harshness in parent-patient interactions (50). However, contrary to our findings, some researchers have found EOI associated with high rates of positive/supportive statements in interaction studies (54).

Regarding relatives’ appraisals of burden in situations of direct and/or recent contact with the patient, an interesting finding emerged thanks to the use of ESM. The item assessing burden was prompted at all assessment points (i.e., independent of whether relatives were with the patient) and also appeared at the end of the questionnaire within the interactional items (i.e., those that relatives only answered if they had direct or recent contact with the patient since the last beep). Of note, both momentary EE dimensions and FQ-criticism, but not FQ-EOI, showed associations with burden when this item was asked in the general part of the questionnaire. However, when answering the same item when in direct or recent contact with the patient, relatives reported negative appraisals as mentioned (being disruptive, makes me feel exhausted), but not feeling burdened. An important difference of this item and the other negative appraisals is that burden is a direct negative assessment of the person, not of behaviors, which may explain the difference in the pattern of responses. This finding underscores the relevance of assessing contextual factors to capture the contextual variability of psychological phenomena.

The following limitations should be acknowledged when interpreting the results. First, our ESM measure of EOI [ESM item: ‘I am worried about (the patient)’] was necessarily brief and concentrated on an essential element but is likely too narrow. Although EOI is characterized by relatives’ over-concern, it is also defined by excessive self-sacrifice, over-identification, and extreme over-protective behavior with the patient (76). Thus, our momentary EOI measure was probably not comprehensive enough to capture the full range of EOI attitudes. Second, it should be noted that we have examined the impact of momentary EE dimensions on momentary emotional and cognitive correlates to investigate the daily life expression of criticism and EOI; nevertheless, given the correlational nature of these data, the opposite interpretation is also plausible (e.g., increased NA contributing to increased reports of momentary EE). Future research should examine whether specific patterns of momentary emotional and cognitive experiences predict the emergence of momentary EE at subsequent assessments using longitudinal designs. Finally, it must be pointed out that the sample size of the present study (n = 55) might be considered relatively small given the number of analyses conducted. However, it must be taken into consideration that it is a substantial sample size in the context of this specific literature. Furthermore, the present study has a sufficient within-person sample based upon the total number of ESM observations. Hox (79) advocates the “50/20 rule” for assessing multilevel data, suggesting that studies should have a minimum of 20 measurements nested within a minimum of 50 participants, which indicates that our study should be adequately powered to test our ESM hypotheses.

The expression of EE dimensions in real time as relatives navigate their real-life settings remains a largely unexplored area of research. This study provided a novel contribution by using momentary as well as psychometric measures to examine the expression of relatives’ EE in relation to a wide variety of real-world experiences. Although criticism and EOI, as measured by momentary and psychometric self-reports, were expected to show relatively differential relationships with daily-life appraisals and caregivers’ subjective states in real-world settings, this study only partly supports the construct validity of momentary EE assessments and the construct and ecological validity of the FQ dimensions. This “low-specificity” pattern of relationships between criticism and EOI with real-world experiences might be related to a developmental issue. The fact that the patients are at an early risk or onset stage of the illness process may indicate that their relatives hyperactivate both the caregiving behavioral system to overprotect the patients, as well as coercive and critical attitudes as an attempt to restore the lost normal behavior and healthy person; these would, respectively, raise EOI and criticism attitudes. It is possible that if the illness progresses and becomes chronic, relatives may start to display a stronger tendency toward either criticism or EOI depending on a complex number of factors pertaining to both the relative (e.g., illness attributions, attachment style) and the patient (e.g., severity, disability).

The present study highlights the utility of ESM for assessing how the predictions derived from EE theory play out in the natural family environment. From a clinical viewpoint, the use of ESM provides a detailed assessment of emotional, cognitive, and behavioral EE components and allows for the critical study of contextual influences such as the caregiver-patient interaction. This enhanced comprehension of the expression of EE in the flow of daily life should facilitate the adaptation of models of EE derived from chronic forms of the disorder to the specificities of early psychosis as well as the development of targeted and personalized interventions, including novel approaches such as ecological momentary interventions (e.g., 80).

## Data Availability Statement

The datasets generated for this study are available on request to the corresponding author.

## Ethics Statement

The studies involving human participants were reviewed and approved by Ethics Committee of the Unió Catalana d’Hospitals (Comitè d’Ètica d’Investigació Clínica (CEIC); number 09-40) and by the Ethics Committee of the Universitat Autònoma de Barcelona (Comissió d’Ètica en l’Experimentació Animal i Humana (CEEAH); number 2679). The participants provided their written informed consent to participate in this study.

## Author Contributions

LH-M contributed to study conception, study design, data collection, data analyses, and writing of the manuscript. TD-M contributed to study conception, study design, data collection and critically revised the manuscript. TK contributed to study conception, study design, provided assistance regarding data analyses and critically revised the manuscript. NB-V was the principal investigator, conceived the study and contributed to study design and writing of the manuscript. All authors have read and approved the final manuscript.

## Funding

This work is supported by the Spanish Ministerio de Economía y Competitividad (PSI2017-87512-C2-01) and the Comissionat per a Universitats i Recerca of Generalitat de Catalunya (2017SGR1612). NB-V is supported by the Institució Catalana de Recerca i Estudis Avançats (ICREA) Academia Award and the Centro de Investigación Biomédica en Red de Salud Mental (CIBERSAM), Instituto de Salud Carlos III, Barcelona, Spain.

## Conflict of Interest

The authors declare that the research was conducted in the absence of any commercial or financial relationships that could be construed as a potential conflict of interest.
